# The Multi-Level Pattern Memory Test (MPMT): Initial Validation of a Novel Performance Validity Test

**DOI:** 10.3390/brainsci11081039

**Published:** 2021-08-05

**Authors:** Elad Omer, Yoram Braw

**Affiliations:** Department of Psychology, Ariel University, Ariel 40700, Israel; eladom@ariel.ac.il

**Keywords:** feigned cognitive impairment, performance validity test (PVT), forced-choice recognition memory, cognitive load, Test of Memory Malingering (TOMM)

## Abstract

Performance validity tests (PVTs) are used for the detection of noncredible performance in neuropsychological assessments. The aim of the study was to assess the efficacy (i.e., discrimination capacity) of a novel PVT, the Multi-Level Pattern Memory Test (MPMT). It includes stages that allow profile analysis (i.e., detecting noncredible performance based on an analysis of participants’ performance across stages) and minimizes the likelihood that it would be perceived as a PVT by examinees. In addition, it utilizes nonverbal stimuli and is therefore more likely to be cross-culturally valid. In Experiment 1, participants that were instructed to simulate cognitive impairment performed less accurately than honest controls in the MPMT (*n* = 67). Importantly, the MPMT has shown an adequate discrimination capacity, though somewhat lower than an established PVT (i.e., Test of Memory Malingering—TOMM). Experiment 2 (*n* = 77) validated the findings of the first experiment while also indicating a dissociation between the simulators’ objective performance and their perceived cognitive load while performing the MPMT. The MPMT and the profile analysis based on its outcome measures show initial promise in detecting noncredible performance. It may, therefore, increase the range of available PVTs at the disposal of clinicians, though further validation in clinical settings is mandated. The fact that it is an open-source software will hopefully also encourage the development of research programs aimed at clarifying the cognitive processes involved in noncredible performance and the impact of PVT characteristics on clinical utility.

## 1. Introduction

The feigning of cognitive impairment is a widespread phenomenon in neuropsychological assessments, especially when these are performed in a medicolegal context [1,2,3]. This jeopardizes the validity of these assessments. Correspondingly, the consensus at present, reinforced by professional organizations (e.g., National Academy of Neuropsychology; NAN), is that evaluating the validity of the examinee’s performance should be an integral part of the neuropsychological assessment process [4,5]. Studies have consistently indicated that subjective clinical judgement is inadequate to detect the feigning of cognitive impairment and other sources of noncredible test performance [6]. This has fueled an ongoing effort to develop validity indicators based either on stand-alone performance validity tests (PVTs) or standard cognitive tests [7]. The former are specifically constructed to detect noncredible performance [8]. In contrast, the latter (i.e., embedded measures) are based on examinee’s performance in existing cognitive tests. These serve a dual purpose: assessing cognitive functioning and detecting noncredible performance [9].

Though existing PVTs received strong empirical support [1,7], there are reasons to continue their refinement and to develop novel PVTs. Examinees may have been exposed to PVTs in previous assessments or acquired information regarding them by other means, such as search engines [10] (pp. 596–597) or legal representatives [11]. With this in mind, we constructed a novel computerized PVT, termed the Multi-level Pattern Memory Test (MPMT). The main impetus for its development was to expand the range of available PVTs. However, the MPMT incorporates several additional advantageous characteristics: (a) A computerized format which allows standardized administration and accurate scoring of the outcome measures. Importantly, the MPMT is based on an open-source software, allowing interested researchers to modify it and explore the impact of different PVT characteristics on clinical utility (see extended description in the Method section). (b) It was constructed to be more cognitively demanding than commonly used PVTs through the use of abstract visual patterns as stimuli and stages that become gradually more challenging. PVTs, and especially those based on the forced-choice recognition memory paradigm, may be identified by examinees as measures of noncredible performance [12]. This may lower their sensitivity and increase their susceptibility to coaching. This risk is likely minimized when using more cognitively challenging PVTs, such as the MPMT. (c) Although verbal PVTs have been translated to various languages (e.g., the Medical Symptom Validity Test, MSVT) [13], the nonverbal stimuli of the MPMT will minimize the challenges inherent in translating and adapting it to different cultures. The use of abstract visual patterns—which are difficult to verbalize—in the MPMT, will also likely decrease cultural bias and the impact of sociodemographic variables. The uniformity of the test stimuli (i.e., each visual pattern consists of an identical number of shapes, similar in size and luminosity) also reduces other sources of variance and will allow easier integration of advanced technologies, such as eye trackers, in future studies [14]. Some of the characteristics described above are incorporated in other PVTs. For example, the Nonverbal Medical Symptom Validity Test (NV-MSVT) uses both nonverbal stimuli and is constructed of subtests, which vary in their difficulty [13,15]. However, we believe that there is still value in the MPMT, considering the need for additional PVTs and its unique characteristics (e.g., options for integrating novel technologies).

We hereby report two experiments which evaluated the MPMT’s utility and examined its performance characteristics. The first experiment compared simulators’ performance in the MPMT to that of honest controls and examined its convergent validity with a well-established PVT. The second experiment replicated the design of the first experiment and analyzed the perceived difficulty of the MPMT’s stages and their association with actual performance. Assessing perceived difficulty is important, as PVTs—which appear more difficult than in actuality—are less transparent and may even encourage an exaggerated impairment profile among those feigning cognitive impairment [16].

## 2. Experiment 1

The experiment aimed to provide an initial evaluation of the MPMT’s utility. We hypothesized that the groups would significantly differ in the MPMT’s primary outcome measure (% correct responses). More specifically, the accuracy of healthy participants instructed to feign cognitive impairment (i.e., simulators) would be significantly poorer than that of honest controls. We also hypothesized that % correct responses would adequately (area under the curve, AUC ≥ 0.8) discriminate between simulators and honest controls, as determined using receiver operating characteristic (ROC) curve analyses. Finally, we hypothesized that there would be strong correlations (*r* ≥ 0.5) between the MPMT’s outcome measures and a well-established PVT, the Test of Memory Malingering (TOMM).

### 2.1. Materials and Methods

#### 2.1.1. Participants

Healthy adults (i.e., 19 to 31 years old) were recruited for the experiment. The inclusion criteria were as follows: (a) age ≥ 18 years; (b) native Hebrew speakers; and (c) intact reading proficiency based on the participants’ self-report. Exclusion criteria included any significant past or current neuropsychiatric disorders. All participants were undergraduate students from Ariel University who performed the experiment as part of their academic requirements. Seventy-two candidates were eligible and willing to participate in the experiment. However, the data of five participants were excluded from analyses (for further details see Data Analysis subsection). Overall, the data of 67 participants were analyzed (sample size was adequate based on power calculations performed using the G*Power 3.1) [17]. The demographic data of the participants are presented in Table 1. This experiment, as well as Experiment 2, were conducted between December 2018 and June 2019. They were approved by the University’s Institutional Review Board (IRB) committee, and all participants provided written informed consent before study participation.

#### 2.1.2. Measures

**Figure 1:**
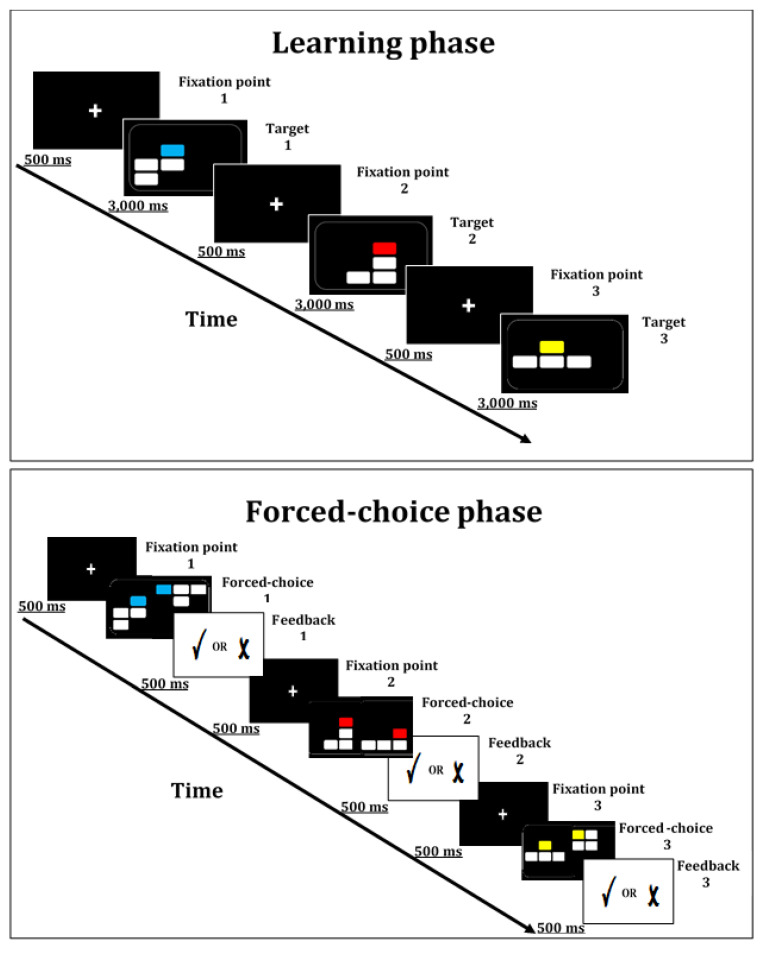
Examples of the learning phase (**top**) and forced-choice recognition phase (**bottom**) of the MPMT.

**Figure 2:**
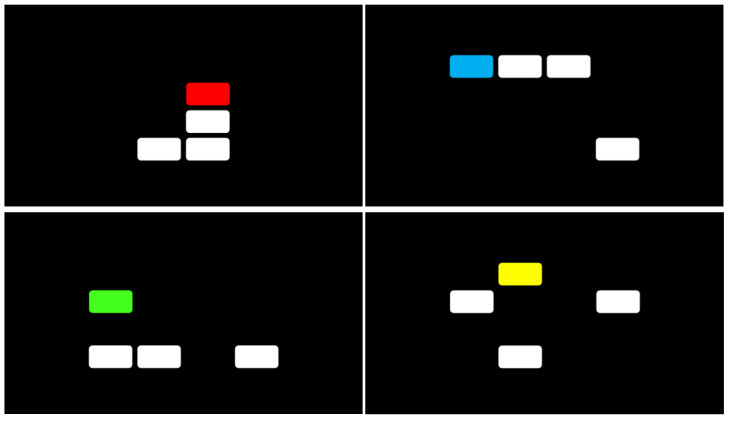
Pattern appearance in the MPMT; representative examples taken from Block 1 (**upper left**), Block 2 (**upper right**), Block 3 (**bottom left**), Block 4 (**bottom right**).

The Multi-level Pattern Memory Test (MPMT) includes a learning phase in which patterns are consecutively presented on a computer screen (3 s per stimulus). Each pattern consists of four rectangles, identical in size (length = 2.5 cm, width = 1.3 cm). These are presented on a 4 × 4 grid with a black background. The rectangles are white, barring one, which is colored (blue, green, red, or yellow). A fixation point is presented during the interstimulus intervals (ISIs) whose duration is 0.5 s. Next, the participant performs a two-choice memory recognition test (target/foil) of each of the previously presented patterns. The target and foil are presented side-by-side, with the location of the target counter-balanced (left or right). Feedback is provided regarding the correctness of each of the participant’s responses. In addition, the MPMT’s stages are divided into blocks and levels. (a) Blocks: The test includes four blocks. In Block 1, all four rectangles are attached (note: although the rectangles are separated by a grid, they seem to be attached to one another due to their proximity; see Figure 1). In Block 2, three rectangles are attached, while the fourth is detached from the rest. In Block 3, two rectangles are attached, while the others are detached. Finally, all rectangles are detached in Block 4. The participant performs the blocks in a fixed order (1 through 4). (b) Levels: Each block is divided into three levels, differing in the number of patterns that the participant is required to memorize (4, 6, and 8). Block 1 consists of consecutive presentation of four patterns (learning stage) followed by a forced-choice recognition test. This constitutes Level 1 of the first block. Next, the participant is required to remember six patterns (Level 2) followed later by Level 3, which requires the participant to remember eight patterns. Thus, the participant is required to remember an increasing number of patterns, likely placing greater demands upon their working memory [18] and accompanied by an expected decrease in performance across levels. In contrast, no change in performance is expected across blocks, as these reflect only variation in spatial organization. As each block is divided into three levels, each block consists of 18 patterns. Thus, the MPMT includes 72 patterns that the participant is required to remember in total (4 blocks × 18 patterns). The test’s total duration is approximately 15 min. It was programmed using Experiment Builder software to control stimulus presentation and response recording [19]; see Figure 1 and Figure 2. Total accuracy (% correct responses) served as the MPMT’s primary outcome measure. Accuracy was also calculated per *block* (i.e., 18 patterns) and *level* (i.e., level 1 = 16 patterns; level 2 = 24 patterns; level 3 = 32 patterns), constituting its secondary outcome measures. The stimuli were presented on an Alienware OptX 23″ display screen with 1920 × 1080 resolution. The eye-to-screen viewing distance was approximately 60 cm. Licensed clinicians can contact the authors for a free-of-charge copy of the MPMT software. Its outcome file is a text file (“.txt”). Participants’ outcome files can then be integrated and analyzed without additional cost, using softwares such as R [20]. However, any alterations of the MPMT characteristics (e.g., changing the number of stimuli) will require purchasing a license from SR Research (Experiment Builder; https://www.sr-research.com/experiment-builder/, accessed on 3 August 2021). As we have a vested interest in promoting the use of the MPMT, we will also do our best to help make such alterations without cost to any researcher interested in using the test. Researchers interested in integrating an eye-tracker with the MPMT will also be required to purchase the Data Viewer software from SR Research, which allows analysis of EDF files (i.e., files containing the participants’ eye movement data).

The Test of Memory Malingering (TOMM) is a forced-choice recognition memory-based PVT in which pictures of common objects are presented (3 s duration per stimulus) over two learning trials, T1 and T2 [21]. Each trial is followed by a two-option forced-choice recognition test, with feedback provided regarding the correctness of the participant’s response. A retention trial may be administered 15 min after completing T2. The TOMM has high specificity and moderate sensitivity, as do other forced-choice recognition based PVTs [22]. T1 accuracy (% correct responses) was used as the TOMM’s outcome measure in the current study, as it was shown to be adequately sensitive. For example, Denning [23,24] found that a T1 total score cutoff of ≤40 (i.e., ≤80% correct responses) was associated with 94% specificity and 72% sensitivity [22].

#### 2.1.3. Procedure

Candidates were notified about the study, including its inclusion and exclusion criteria (see Participants subsection), using the departmental electronic research bulletin board. Eligible candidates filled out a demographic–medical questionnaire and were randomly assigned to either a simulation (SIM: *n* = 33) or honest control (HC: *n* = 34) condition. The simulators were provided with a script and requested to play the part of an examinee who was involved in a car accident in which they sustained a concussion. Despite returning to previous levels of functioning, the examinee has decided to falsify the existence of cognitive impairment, thereby receiving a larger settlement in a lawsuit filed against their insurance company. Simulators were requested not to overexaggerate their impairment so as not to raise suspicions regarding its authenticity [16] (pp. 592–614) and were notified that they would be entered into a raffle with a chance to win ILS 350 upon successfully avoiding detection. Honest controls, in contrast, were requested to perform the tests to the best of their ability. Next, all participants performed the MPMT and TOMM T1 in a pseudo-randomized order.

We used two a priori rules for evaluating the participants’ compliance with the experimental instructions: (a) After completing the experimental procedures, participants filled out a manipulation check questionnaire indicating their motivation to perform the tasks as instructed. This element of the manipulation check is termed “reported effort” by [10] (pp. 599–600). A score <4 on a 7-point Likert scale was selected for exclusion of participants’ data. (b) TOMM T1 performance of honest controls that was below the selected cutoff (i.e., ≤80% correct responses) was deemed extremely poor and unrepresentative of the expected cognitive functioning of honest undergraduate responders. The data of these participants were, therefore, discarded from analyses.

#### 2.1.4. Data Analysis

*Preliminary procedure*: All participants reported adequate compliance with experimental procedures in the manipulation check questionnaire. However, the data of one honest control were excluded from analyses due to extremely poor TOMM T1 performance (58% correct responses). In addition, four simulators also had exceptionally low scores (TOMM T1 = 2% correct responses; significantly below-chance performance). Though not specified in our a priori rules for excluding data, their performance was considered unrepresentative of real-life performance of those feigning cognitive impairment. Their data were, therefore, excluded from analyses. Altogether, the data sets of five participants were excluded from analyses.

*Group comparisons and classification accuracy*: The groups were compared in demographic variables and in the primary outcome measures of the MPMT and TOMM T1 (% correct responses), using independent samples t-tests and chi-square analyses (for parametric and non-parametric measures, respectively). For these analyses, as well as those described henceforth, statistical significance was set at *p* < 0.05. ROC curve analyses were used to assess the discrimination capacity of the MPMT’s primary outcome measure. The ROC curve is a plot of true positive rates (sensitivity) against false positive rates (i.e., 1-specificity). The AUC has a range of 0.5 to 1.0 and is an expression of the group discrimination capacity (larger values indicate better discrimination capacity). Qualitative interpretation of the AUCs of the ROC curves was performed in accordance with [25]; poor (0.60–0.69), acceptable (0.70–0.79), excellent (0.80–0.89), and outstanding (≥0.90). Next, cutoffs for the MPMT’s primary outcome measure that maintained high specificity (≥90%; i.e., false positive rate of ≤10%) and sensitivity of ≥50% for the detection of noncredible performance were chosen. This was done in accordance with the conventions in the field [2]. The general guidelines of [26] were then used to qualitatively interpret sensitivity values (see p. 32); low (<40%), moderate (40–69%), and high (≥70%). Additionally, we calculated the positive and negative predictive power (PPP and NPP, respectively). PPP is an index of the probability of accurately identifying noncredible responders. It is calculated by dividing true-positives by the sum of true- and false-positives. NPP pertains to the probability of accurately identifying credible responders. It is calculated by dividing true-negatives by true- and false-negatives. Both PPP and NPP utility estimates take base rates into account and therefore are instrumental in informing clinical decisions in real-life settings [27,28]. Estimated base rates of noncredible performance in medicolegal settings [3,29] were used to calculate PPP and NPP values in the current study [5]. 

*Reliability and validity*. The internal consistency of the MPMT (i.e., the extent to which its items measure aspects of the same construct) was assessed using Cronbach’s alpha coefficient. Convergent validity was established using a two-pronged approach: (a) We performed Pearson product–moment correlations between the primary outcome measures of the MPMT and TOMM T1. This was done for the overall sample, as well as separately for each group. (b) We performed a ROC curve analysis of the MPMT’s primary outcome measure and used a z-test to compare it to that of the TOMM T1 outcome measure [30]. In addition, the sensitivity and specificity values of the TOMM T1 were established, based on its suggested cutoff (≤80% correct responses indicating noncredible performance). These were qualitatively compared to those of the MPMT’s primary outcome measure, derived using the ROC curve analysis.

### 2.2. Results

**Figure 3:**
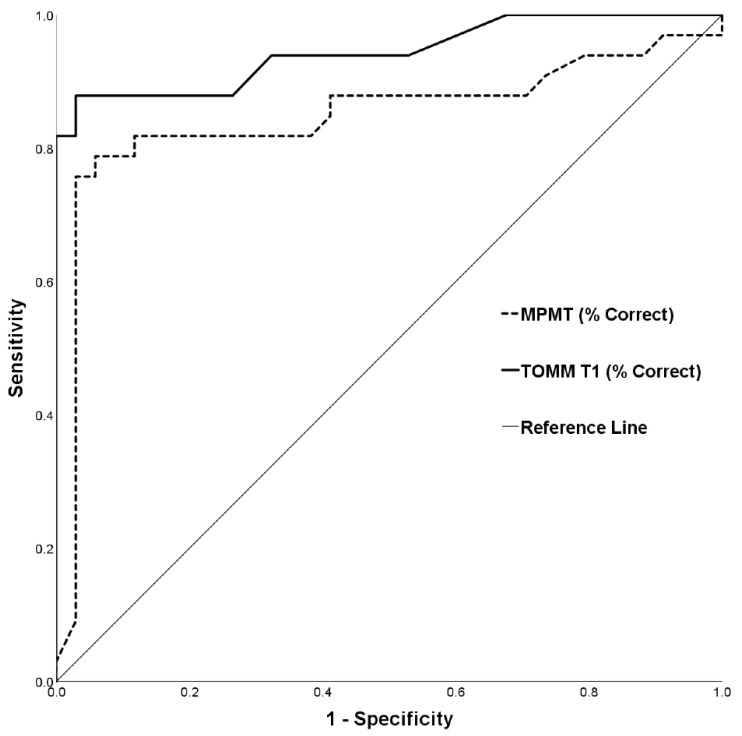
ROC curves for differentiating simulators and honest controls using the MPMT (AUC = 0.85) and TOMM T1 (AUC = 0.94). Note: AUC = Area under the curve; ROC = receiver operating characteristic; TOMM T1= Test of Memory Malingering trial 1.

*Group comparisons and classification accuracy*. As expected, simulators made significantly fewer correct responses than honest controls in both the MPMT and TOMM T1 (*p* < 0.001; see Table 1). Notably, the honest controls tended to be less accurate in the MPMT than the TOMM T1 (73.3% vs. 94.9%). The high accuracy of the participants in the TOMM T1 corresponds to those of previous studies of the TOMM [21] and other PVTs with a similar methodology [31,32]. Based on the ROC curve analysis, the MPMT’s primary outcome measure (% correct responses) had an excellent discrimination capacity (AUC = 0.85, *p* < 0.001, 95% CI: 0.74–0.95); see Figure 3. Perfect specificity, though achievable, was associated with negligible sensitivity (2.9%). In contrast, a 54.9% cutoff was associated with excellent specificity (97.2%) and 76.5% sensitivity, while a 59.4% cutoff maintained adequate specificity (91.7%) with slightly enhanced sensitivity (79.4%). Cutoffs and associated utility estimates for the MPMT’s primary outcome measure are presented in Table 2. Note that PPP values associated with cutoffs ≤ 53.5% correct responses have a U-shaped curve. This is unexpected and is due to one honest control, who scored 37.5% in the MPMT, leading to instability in the data set and stressing the need for further research to establish suitable cutoffs for the MPMT.

*Reliability and validity*. Cronbach’s alpha coefficient was 0.89 for the MPMT’s primary outcome measure. Regarding convergent validity, the primary outcome measures of the MPMT and TOMM T1 were strongly correlated, both in the overall sample (*r* = 0.740, *p* < 0.001) and within each group (SIM: *r* = 0.560, *p* = 0.001; HC: *r* = 0.525, *p* = 0.001). Only one honest control failed the TOMM T1 (specificity = 97.1%), while six simulators scored in the valid range of the TOMM T1 (sensitivity = 81.8%), corresponding to earlier studies of the TOMM [1]. When equating cutoffs with similar specificity values between the two PVTs (i.e., specificity of 97.1% in the TOMM T1 and 97.2% in the MPMT), the MPMT was slightly less sensitive than the TOMM T1 (76.5% and 81.8%, respectively). The TOMM’s excellent classification was also mirrored in its outstanding discrimination capacity (AUC = 0.94, *p* < 0.001, 95% CI: 0.89–1.00), stronger than that of the MPMT (*z* = 2.19, *p* = 0.028). Regarding clinical utility, however, this difference was manifested in only two additional correct classifications by the TOMM T1 (i.e., one participant in each group).

## 3. Experiment 2

This experiment replicated the design of the first experiment, while also analyzing the participants’ subjective experience of cognitive load and its association with their performance in the MPMT. More specifically, the MPMT utilizes a hierarchical approach, also termed profile analysis, which has shown promise for the detection of noncredible performance [34]. It consists of analyzing the participants’ performance across the MPMT’s blocks and levels, predicting a dissociation between the performance profiles of the two groups. As in the first experiment, we hypothesized that the MPMT’s primary outcome measure (% correct responses) would adequately (AUC in ROC curve analyses ≥0.8) discriminate between simulators and honest controls. We also hypothesized that the simulators’ performance would consistently decline across both blocks and levels as the test progresses. That is, simulators’ accuracy (% correct responses) was hypothesized to significantly decrease across both blocks (1 to 4) and levels (1 to 3). In contrast, we hypothesized that honest controls’ accuracy (% correct responses) would decline across the MPMT’s levels (1 to 3), but not across its blocks (i.e., performance would be stable across blocks 1 to 4). Note that the former, but not the latter, are likely associated with increased cognitive load; see description of the MPMT. Finally, we hypothesized that the MPMT would be perceived by both simulators and honest controls as progressively becoming more challenging across both blocks (1 to 4) and levels (1 to 3). In other words, a dissociation was expected between actual performance in the MPMT and the participants’ subjective experience of cognitive load, stemming from performing the MPMT. 

### 3.1. Materials and Methods 

#### 3.1.1. Participants

Data of 78 healthy undergraduate students (18 to 38 years old) were included in the experiment, partially overlapping with that included in [35]. Inclusion and exclusion criteria were the same as in the first experiment. The data of one participant were discarded due to technical problems (final *n* = 77). The demographic data of participants are presented in Table 3. 

#### 3.1.2. Measures

Mental Effort Rating Scale (MERS). This is a self-report questionnaire in which participants rate their subjective experience of cognitive load [36]. More specifically, participants are requested to report their invested mental effort on a 9-grade symmetrical categorical scale, with high scores indicating greater mental effort. In the current experiment, the scale was presented to participants after completing each level of the MPMT with the following instruction: “Please rate the mental effort you experienced while performing the test”. The mean MERS score (i.e., mean ratings of all MERS items) was calculated, as well as separate MERS scores for each block (1 through 4). The latter were calculated by averaging the scores of the three levels that comprised each block. 

*MPMT*. The test was identical to that used in the first experiment, except for a screen that appeared after completing each level in which participants were requested to fill out the MERS.

#### 3.1.3. Procedure

Eligible candidates, notified about the study using the departmental electronic research bulletin board, filled out a demographic–medical questionnaire and were then randomly assigned to either a simulation (SIM: *n* = 39) or honest control condition (HC: *n* = 38), as in the first experiment. Next, they performed the MPMT while filling out the MERS after each level (i.e., 12 times). Finally, they filled out a manipulation check questionnaire.

#### 3.1.4. Data Analysis

The discrimination capacity of the MPMT primary outcome measure (% correct responses) was assessed using a ROC curve analysis. A two-way repeated-measures analysis of variance (ANOVA) was then performed on the MPMT’s secondary outcome measures (% correct responses in each block and level). It included a between-subjects factor of *group* (SIM/HC) and within-subject factors of *block* (1/2/3/4) and *level* (1/2/3). Post-hoc comparisons were performed using paired-samples t-tests. The participants’ perceived cognitive load (i.e., MERS scores) across the MPMT’s blocks and levels was then analyzed using a similar repeated-measures ANOVA. 

### 3.2. Results

**Figure 4:**
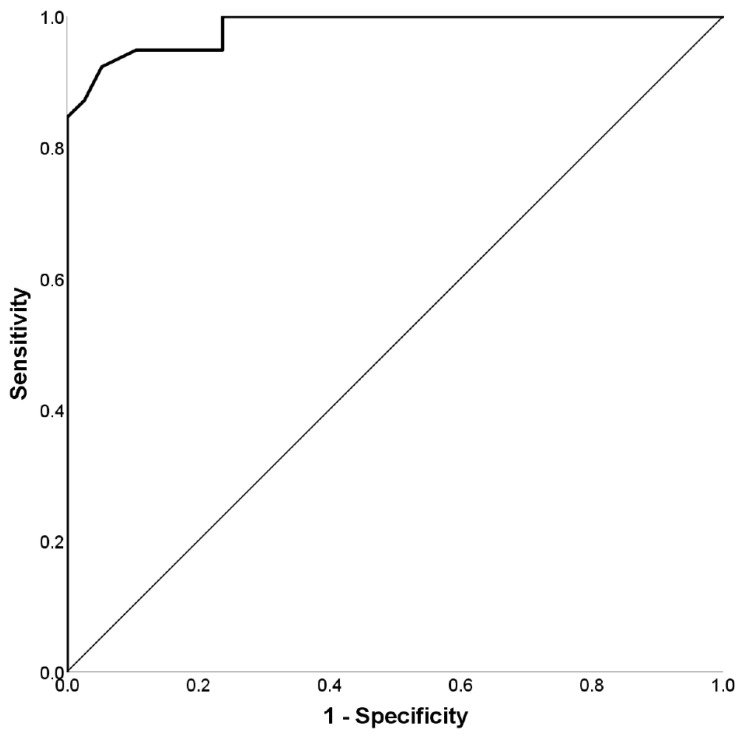
ROC curves for differentiating simulators and honest controls using the MPMT (AUC = 0.98). Note: AUC = area under the curve; ROC = receiver operating characteristic.

Accuracy (% correct responses) in the MPMT had an outstanding discrimination capacity (i.e., stronger than that found in the first experiment) (AUC = 0.98, *p* < 0.001, 95% CI: 0.96–1.00); see Figure 4. 

**Figure 5:**
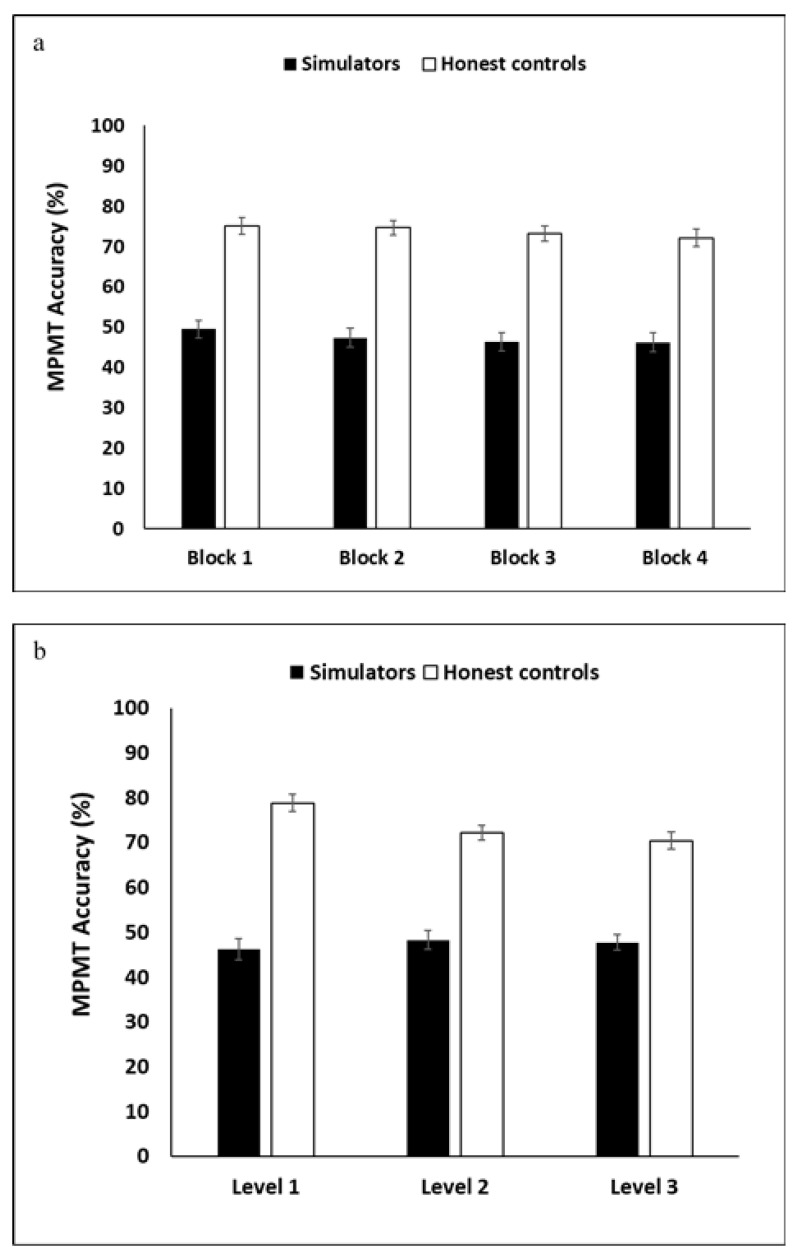
Accuracy scores per group across the four MPMT blocks (**a**) and three levels (**b**).

Correspondingly, the simulators performed more poorly than honest controls in the MPMT, as is evident by the ANOVA’s significant *group* main-effect (*F*(1,75) = 168.87, *p* < 0.001, η*_p_*² = 0.69). Importantly, the *group* × *level* interaction was significant (*F*(2,150) = 5.78, *p* = 0.004, η*_p_*² = 0.07). Post-hoc analyses indicated that the accuracy of honest controls declined between levels 1 and 2 (*t*(37) = 2.55, *p* = 0.015) and between levels 1 and 3 (*t*(37) = 4.21, *p* < 0.001), but did not significantly change between levels 2 and 3 (*p* = 0.383). In contrast, the performance of simulators essentially did not change across the levels. The *block* and *level* main effects were not significant (*p* = 0.352, *p* = 0.095; respectively). No other significant main effects or interactions were found in the ANOVA; see Figure 5.

**Figure 6:**
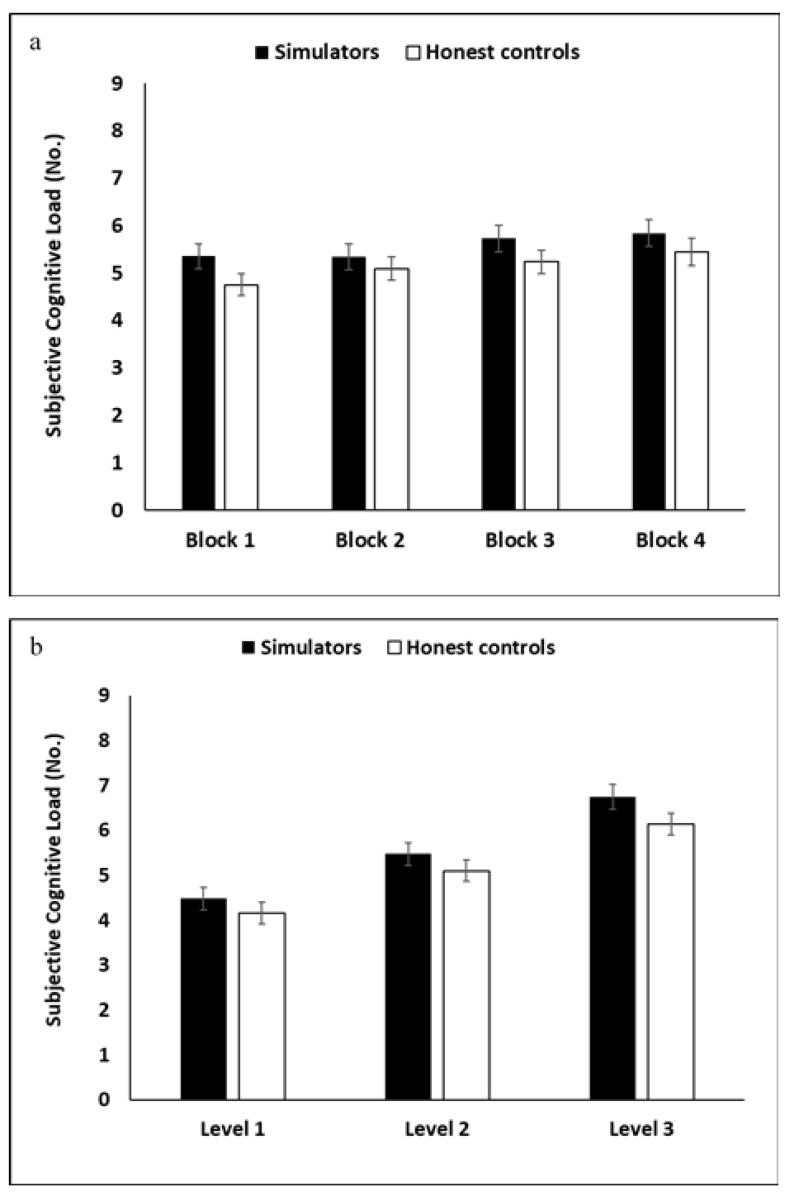
Subjective cognitive load ratings per group across the four MPMT blocks (**a**) and three levels (**b**).

An analysis of participants’ perceived cognitive load revealed significant *block* and *level* main-effects (*F*(3,225) = 6.50, *p* < 0.001, η*_p_*² = 0.08; *F*(2,150) = 154.43, *p* < 0.001, η*_p_*² = 0.67; respectively); participants reported experiencing more cognitive load as they progressed through the MPMT. The only significant interaction was between *block* and *level* (*F*(6,450) = 2.41, *p* = 0.026, η*_p_*² = 0.03). The *group* main effect was not significant (*p* = 0.189); see Figure 6.

## 4. General Discussion

The study assessed the utility of a novel computerized forced-choice recognition memory-based PVT, termed the MPMT. Aside from extending the range of PVTs at the disposal of clinicians, the MPMT has several additional merits. These include a more cognitively demanding format than commonly used PVTs (likely minimizing its transparency for examinees) and the use of abstract visual patterns, which is aimed at minimizing cultural bias. It is also based on an open-source software, allowing researchers to use it free of charge. Manipulating key variables (e.g., number of stimuli/foils, length of presentation, etc.) allows researchers to devise research programs aimed at enhancing our understanding of factors affecting PVT performance and utility. Note, however, that such alterations require purchasing a licensed software from SR Research or being aided by the authors of the current study, as detailed in the Measures subsection. Two experiments were performed with the purpose of providing initial evaluation of the MPMT and to examine its performance characteristics. As part of the first experiment, we compared the performance of simulators and honest controls in the MPMT and assessed its convergent validity with a well-established PVT (TOMM). Experiment 2 was a replication study which further evaluated the MPMT’s components. More specifically, we assessed the participants’ objective performance and subjective experience of cognitive load, investigating the utility of a hierarchical approach for the interpretation of examinees’ MPMT performance.

As expected, simulators performed the MPMT significantly more poorly than honest controls in Experiment 1. The MPMT’s primary outcome measure (% correct responses) was also a strong predictor of group membership, as is evident in ROC curve analysis (AUC = 0.85) and the adequate sensitivity and specificity values that were reached in the first experiment. For example, a 59.4% accuracy cutoff was associated with 91.7% specificity and 79.4% sensitivity. Thus, the MPMT proved highly effective in differentiating simulators from honest controls. Correspondingly, the MPMT also showed adequate convergent validity, reflected in the strong correlation of its primary outcome measure with that of the TOMM T1 (*r* = 0.740). This key finding indicates that the two PVTs measure a similar construct, while suggesting that administrating both the MPMT and other two-choice forced-choice recognition memory-based PVTs in neuropsychological assessments may be redundant. Two additional findings are notable in this regard. First, the MPMT had a somewhat weaker discrimination capacity than the TOMM T1, as is evident when comparing their ROC curves. This is also evident when comparing cutoffs derived from the two PVTs, which are associated with similar specificity values. For example, the TOMM T1 was slightly more sensitive than the MPMT (81.8% and 76.5%, respectively) when comparing cutoffs with similar specificity values (TOMM T1: 97.1%; MPMT: 97.2%). These statistical differences, however, may have limited clinical ramifications, as only two additional participants were correctly classified by the TOMM T1. Second, the MPMT seems to be more cognitively challenging than the TOMM and perhaps other forced-choice recognition memory-based PVTs. This is evident in the lower accuracies of the honest controls in the MPMT compared to the TOMM T1 (73.3% vs. 94.9%). Correspondingly, high specificity values (i.e., ≥90%) were attained with lower accuracy cutoffs in the MPMT (≤59.4%) compared to other PVTs with similar methodology (e.g., the TOMM and WMT) [21,32]. Consequently, the MPMT is less likely to be perceived as a PVT (i.e., low face validity), reducing the possibility that those attempting to feign impairment will enact counter measures [37]. This finding also suggests that it may serve a dual purpose: to assess credibility and to examine cognitive functioning, similar to the subtests incorporated in the WMT (i.e., Multiple Choice, Paired Associates, Free Recall and Long Delayed Free Recall subtests) [32]. At the same time, those with genuine cognitive impairment may be at an increased risk of being classified as false positives in the MPMT, emphasizing the need for further studies in clinical settings. 

Experiment 2 replicated the findings of the first experiment. The MPMT showed outstanding discrimination capacity, as is evident in the ROC curve analysis of its primary outcome measure (AUC = 0.98). As expected, simulators performed the MPMT significantly more poorly than the honest controls. Importantly, an analysis of participants’ performance revealed a significant interaction between the experimental group and the MPMT’s levels. The performance of honest controls deteriorated as they were required to remember more items (i.e., 4, 6, and 8 patterns), an expected finding considering the greater demands that were placed upon their working memory [18] as the levels progressed. Note in this regard that the blocks reflected only variations in spatial organization and were therefore not expected to differ meaningfully in their cognitive load. In contrast, the performance of simulators did not differ significantly as a function of either the MPMT’s levels or blocks. A dissociation was, therefore, found between their objective performance and subjective ratings of task difficulty. This was surprising, as the simulators—like the honest controls—perceived the test as becoming more difficult with each block and level. A likely explanation for this finding (i.e., the accuracy did not decline in tandem with their perception of task difficulty) is a floor effect in the simulators’ performance. According to this explanation, further decline in performance was not possible due to the poor performance at the beginning of the task, insufficient adjustment of performance as the task progressed, or some combination of the two. 

The above-mentioned performance profile of simulators in the MPMT suggests the usefulness of a detection method referred to as a *performance curve* [16] (pp. 27–31), also known as a hierarchical approach. It is based on the general finding that genuine examinees produce a predictable performance pattern (i.e., increased item difficulty is associated with poorer performance). In contrast, those feigning cognitive impairment, unaware of this expected performance pattern or unable to adequately adjust their performance, exhibit a flat performance curve in which their performance in easy and difficult items is similar. For example, simulators may perform more poorly than dementia patients on the two primary performance validity subtests of the WMT (i.e., immediate and delayed recognition: IR and DR, respectively), while achieving better scores than these patients on its more difficult subtests which are sensitive to memory impairment [32]. For additional information regarding the utility of the hierarchical approach as a detection method, see recent studies [34,38]. 

Several limitations of the current study should be noted. First, simulation studies provide excellent control over internal validity and are, therefore, suited for exploratory research. However, the external validity of this research design has been criticized [10] (p. 11) and this is especially notable as the MPMT appears to be more cognitively challenging than other forced-choice recognition memory-based PVTs. Additionally, the current study’s sample was comprised of healthy undergraduate students. This should be taken into consideration as they may not necessarily perform the tests as requested (e.g., honest controls may not perform to the best of their ability). Note in this regard that the issue of suboptimal effort among these participants is of concern and has received attention in previous studies [39]. There is, therefore, a need for studies in real-life clinical settings to ensure the generalizability of the findings and to confirm that false positives do not exceed the conventions in the field [2]. The inclusion of clinical patients (e.g., traumatic brain injury patients) in simulation studies will clarify whether simulators can be differentiated from examinees with neuro-psychiatric disorders when performing the MPMT, rather than from healthy controls (as in the current study). The use of a known group’s research design in future studies is especially recommended. It is based on highly accurate classifications of “known groups” in clinical settings, according to established criteria [10] (pp. 11–12). Researchers are also encouraged to characterize the performance curve of different clinical populations in the MPMT, as the use of profile analysis is a particularly appealing feature of the MPMT [34,38]. Second, convergent validity was assessed using only one PVT (i.e., TOMM). Further research is needed to validate the findings using other PVTs, as well as validity indicators based on standard neuropsychological tests (i.e., embedded measures). Finally, as the MPMT is likely more cognitively challenging than other PVTs, it would be of interest to assess whether examinees perceive it as a measure of noncredible performance (i.e., its face validity). This is important considering the threats posed to PVTs by information which is available online [40]. Relatedly, investigating the discrimination capacity of the MPMT’s levels may be helpful in clarifying whether they differ in utility. It would be particularly interesting to examine whether the first level is associated with enhanced specificity compared to levels 2 and 3 (which are presumably more cognitively demanding), possibly indicating a path to decrease the test administration time (i.e., administering only one level instead of three). Regarding future studies, we encourage researchers to explore theoretical questions regarding PVT characteristics that enhance their clinical utility by, for example, altering the number of blocks/levels, length of stimuli presentation, etc. This will require purchasing a software license from SR Research (Experiment Builder) or being aided by the authors of the current study, as detailed earlier. Additionally, investigating the utility of response time data provided by the MPMT, which was not the focus of the current study, would be of interest considering the initial evidence regarding its potential [41]. It should also be noted that the MPMT was constructed to allow easy integration with eye-tracking technology [14]. For example, target and foil pairs have an identical number of rectangles and luminosity. The use of an eye-tracker is, therefore, another promising path which researchers may consider following. More research is also needed regarding the MPMT’s reliability and validity. This is especially notable regarding construct validity, as it likely differs from other forced-choice memory-based PVTs, due to its more cognitively challenging nature. Researcher examining MPMT performance of older adults should take into account that participants may differ in their technology proficiency (e.g., use of computerized tasks) due to factors such as age [42,43]. Researchers are, therefore, advised to consider the impact of this factor (e.g., assess participants’ age and technology proficiency) on their findings. Finally, when planning future studies, researchers should note the inherent challenges in performing power analyses of within subjects designs (e.g., analyses of performance across the MPMT levels and blocks) and should consult the relevant literature when performing required sample size calculations [44].

## 5. Conclusions

The current study’s findings provide initial validation of a novel computerized PVT. The MPMT proved effective in differentiating simulators from honest controls, and cutoffs derived from its primary outcome measure were associated with satisfactory utility estimates. It also showed adequate convergent validity with the TOMM, a well-established PVT. Its relatively cognitively demanding nature and structure reduce its transparency (i.e., low face validity as a PVT) and allow profile analysis. Its open-source setup and amenability to alterations by researchers (see Measures subsection for additional information) make it highly suitable to explore the utility of its features (e.g., length of stimuli presentation), which will likely enhance its clinical efficacy. Hopefully, these characteristics will encourage validation of the current study’s preliminary findings and exploration of both theoretical and practical aspects of noncredible performance detection. Further research with different neuropsychiatric populations is a necessary first step in exploring the MPMT’s potential.

## Figures and Tables

**Table 1:** Demographic and outcome measures of the TOMM and MPMT among simulators (*n* = 33) and honest controls (*n* = 34).

Measures		Simulators (SIM)	Honest Controls (HC)	Statistical Analyses	Post-Hoc Tests	Effect Size
Demographic	Age (years) [mean ± SD (range)]	23.38 ± 2.26 (20–31)	22.68 ± 1.79 (19–27)	*t*(65) = −1.41, *p* = 0.162	____	*d* = 0.34
	Education (years) [mean ± SD]	12.00 ± 0.25	11.97 ± 0.17	*t*(65) = −0.56, *p* = 0.575	____	*d* = 0.14
	Gender (women) [number (%)]	26 (78.80%)	31 (91.20%)	*χ^2^*(1) = 2.02, *p* = 0.155	____	*OR* = 0.36
TOMM T1	Accuracy (% correct)	57.45 ± 21.15	94.88 ± 5.53	*t*(65) = 9.97, *p* < 0.001	HC > SIM	*d* = 2.42
MPMT	Total accuracy (% correct)	53.17 ± 14.21	73.30 ± 10.76	*t*(65) = 6.55, *p* < 0.001	HC > SIM	*d* = 1.60

Note: TOMM T1 = Test of Memory Malingering trial 1.

**Table 2:** Classification accuracy based on utility estimates (specificity/sensitivity/PPP/NPP values) of the MPMT’s primary outcome measure (% correct responses).

Cutoff (%)	Specificity (%)	Sensitivity (%)	PPP (%)			NPP (%)		
			Base Rates			Base Rates		
			20%	30%	40%	20%	30%	40%
48.2	97.2	41.2	78.7	86.4	90.8	86.9	79.4	71.3
48.9	97.2	47.1	80.9	87.9	91.9	88.0	81.1	73.4
49.7	97.2	52.9	82.7	89.1	92.7	89.2	82.8	75.6
50.4	97.2	55.9	83.4	89.6	93.1	89.8	83.7	76.8
51.4	97.2	64.7	85.3	90.9	94.0	91.7	86.5	80.5
52.4	97.2	67.6	85.9	91.3	94.2	92.3	87.5	81.8
53.5	97.2	73.5	86.9	91.9	94.6	93.6	89.6	84.6
54.9	97.2	76.5	87.3	92.2	94.8	94.3	90.6	86.1
56.0	94.4	76.5	77.5	85.5	90.2	94.1	90.4	85.8
57.3	94.4	79.4	78.1	86.0	90.5	94.8	91.5	87.3
59.4	91.7	79.4	70.4	80.3	86.4	94.7	91.2	87.0
60.8	88.9	79.4	64.1	75.4	82.7	94.5	91.0	86.6
61.8	86.1	79.4	58.8	71.0	79.2	94.4	90.7	86.3
63.2	86.1	82.4	59.7	71.8	79.8	95.1	91.9	88.0

Note: Cutoffs presented with sensitivity ≥40% (considered moderate-and-high values) [26] and specificity ≥84%. The latter was suggested as the lowest acceptable specificity for validity indicators [33], though 90% was established over the years as a more appropriate specificity threshold [1,2,9,26]. NPP = negative predictive power; PPP = positive predictive power.

**Table 3:** Demographic and outcome measures of the MPMT among simulators (*n* = 39) and honest controls (*n* = 38).

Measures		Simulators (SIM)	Honest Controls (HC)	Statistical Analyses	Effect Size
Demographic	Age (years) [mean ± SD (range)]	23.79 ± 3.48 (18–38)	23.00 ± 2.01 (20–28)	*t*(75) = −1.22, *p* = 0.225	*d =* 0.28
	Education (years) [mean ± SD]	12.46 ± 0.91	12.21 ± 0.70	*t*(75) = −1.35, *p* = 0.182	*d =* 0.31
	Gender (women) [number (%)]	32 (82.05%)	36 (94.73%)	*χ^2^*(1) = 3.00, *p* = 0.083	*OR* = 0.25

## Data Availability

The data that support the findings of this study are available from the corresponding author upon reasonable request.
